# Gliclazide protects ionizing radiation-induced intestinal injury in mice by inhibiting oxidative stress and caspase-3

**DOI:** 10.5114/bta.2024.145257

**Published:** 2024-12-19

**Authors:** Soroush Arzani, Soghra Farzipour, Fereshteh Talebpour Amiri, Seyed Jalal Hosseinimehr

**Affiliations:** 1 Department of Radiopharmacy, Faculty of Pharmacy, Mazandaran University of Medical Sciences, Sari, Iran; 2 Student Research Committee, Mazandaran University of Medical Sciences, Sari, Iran; 3 Department of Anatomy, Faculty of Medicine, Mazandaran University of Medical Sciences, Sari, Iran

**Keywords:** gliclazide, caspase-3, oxidative stress, intestinal injury, radioprotective, ionizing radiation

## Abstract

Gliclazide (GLZ), an oral antihyperglycemic medication, has additional beneficial effects, such as anti-inflammatory and antioxidant properties, besides lowering blood glucose levels. In this study, the radio-protective effect of GLZ was evaluated against ionizing radiation (IR)-induced intestinal injury in mice. Eight groups of mice were randomized as follows: control, GLZ (5, 10, and 25 mg/kg), IR (6 Gy), and IR + GLZ (at 5, 10, and 25 mg/kg). GLZ was administered to the mice for eight consecutive days, after which they were exposed to X-rays at a single dose of 6 Gy. After irradiation, biochemical parameters, immunohistochemical, and histological examinations were conducted on the ileum of the mice. IR exposure increased the levels of malondialdehyde and protein carbonyl, while glutathione levels, as oxidative stress biomarkers, decreased. Apoptosis in ileum tissues was also assessed. Furthermore, histopathological changes were observed in the irradiated mice. GLZ treatment significantly mitigated these changes. The administration of GLZ resulted in a marked decrease in caspase-3 immunoreactivity in the ileum of irradiated mice. This preclinical study exhibited that GLZ has a radioprotective effect against intestinal injury by inhibiting oxidative stress and apoptosis.

## Introduction

Pelvic radiotherapy treats tumors in the abdominal region, including those affecting the gastrointestinal, urological, or gynecological systems. However, radiotherapy has several side effects that limit its application. The gastrointestinal tract is particularly radiosensitive. Ionizing radiation (IR) induces gastrointestinal injuries, leading to a reduction in patients' quality of life. Pelvic irradiation affects normal tissues, disrupting physiological functions and causing side effects like diarrhea, incontinence, tenesmus, rectal bleeding, radiation proctitis, and radiation cystitis (Birgisson et al., 2007; Morris and Haboubi, 2015; Klopp et al., 2018). IR increases reactive oxygen species (ROS) levels and triggers inflammation in surrounding tissues (Kim et al., 2012; Moussa et al., 2016). Accumulated ROS in intestinal tissue elevates oxidative stress biomarkers (Musa et al., 2019), leading to intestinal dysfunction. Damage to crypt cells in the intestinal epithelium, reduced number and size of villus crypts, ulcers, and necrosis are common outcomes. Cells with high mitotic activity, like those in the intestinal epithelium, are particularly sensitive to IR-induced damage (Akpolat et al., 2020). An increased level of caspase-3 expression, a biomarker of apoptosis, has been observed in intestinal crypt cells of irradiated rats and is associated with intestinal mucosal injury (Akpolat et al., 2020). Oxidative stress and inflammation are the two main mechanisms involved in IR-induced intestinal injury. Various radioprotective agents, such as mefenamic acid, Zataria extract, and oxymetholone, have been evaluated for their antioxidant, anti-inflammatory, and bone marrow regeneration effects in animal models to mitigate IR-induced side effects (Hosseinimehr et al., 2006; Hosseinimehr, 2007; Hosseinimehr et al., 2011; Hosseinimehr et al., 2015).

Gliclazide (GLZ), a second-generation sulfonylurea, is used to control blood glucose levels in diabetic patients (Alper et al., 2005). GLZ can reduce free radical generation, and lipid peroxidation, and increase the levels of antioxidant enzymes like superoxide dismutase and endogenous thiols in diabetic patients at therapeutic doses (O’Brien et al., 2000). It has also protected healthy human lymphocytes against oxidative stress by decreasing ROS-induced DNA damage (Sliwinska et al., 2008). Additionally, GLZ has been reported as a radioprotective agent against IR-induced chromosome damage due to its antioxidant activity (Pouri et al., 2019). Besides its antioxidant property, GLZ significantly decreases the levels of IL-6, a pro-inflammatory mediator, while increasing IL-10, an anti-inflammatory cytokine, in healthy colon tissues, mitigating ulcerative colitis (Arafa et al., 2020). GLZ has also been shown to attenuate cisplatin-induced nephrotoxicity and hepatotoxicity by inhibiting oxidative stress and inflammation in mice (Taghizadeh et al., 2020; Taghizadeh et al., 2021). In this study, we evaluated the radioprotective effect of GLZ against IR-induced ileum injury and apoptosis in mice.

## Materials and methods

### Reagents

GLZ was procured from Tehran Daru (Iran) at pharmaceutical grade. The primary antibody, caspase-3, was obtained from Abcam (Lat: ab184787), and the secondary antibody (Mouse and Rabbit Specific HRP/DAB) was from Abcam (Lat: ab64264). Other reagents and solvents were purchased from Sigma (USA) and Merck (Germany).

### Animals and experimental protocol

Sixty-four male BALB/c mice weighing 25–30 g were obtained from the animal lab of Mazandaran University of Medical Sciences Research Center (Sari, Iran). The animals were housed under standard conditions (temperature 25 ± 2°C, humidity 55 ± 5%, and a 12-h light-dark cycle) for 1 week before the start of the experiment. The mice had free access to water and food. This experimental animal study was approved by the Ethics Committee of the University (ID#IR.MAZUMS.REC.1400. 8619) and reported according to the ARRIVE guidelines (Animal Research: Reporting of In Vivo Experiments). The animals were randomly distributed into eight groups (eight mice in each group) as follows:

- control group: mice received distilled water orally for 8 consecutive days;
- GLZ groups: mice were administered GLZ at doses of 5, 10, and 25 mg/kg orally for 8 consecutive days;
- IR group: mice were exposed to whole-body X-ray irradiation at a dose of 6 Gy;
- GLZ + IR groups: mice were administered GLZ at doses of 5, 10, and 25 mg/kg orally for 8 consecutive days and then exposed to X-ray at a single irradiation dose of 6 Gy;
- GLZ (at doses 5, 10, and 25 mg/kg) was suspended in distilled water and administrated into mice through oral gavage.

The GLZ doses in this study were chosen based on previous studies (Taghizadeh et al., 2020, 2021). The experimental schedule was adapted from a previous study on lung tissue with brief modifications (Farzipour et al., 2020).

### Irradiation of mice

**Fig. 1:**
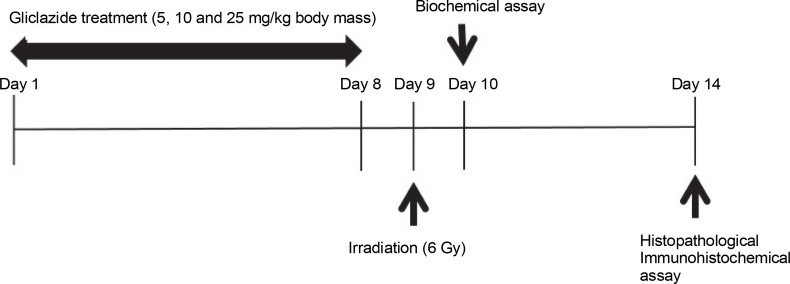
Study design diagram for evaluating the effect of gliclazide on ileum damage induced by ionizing radiation

A Plexiglas box with 18 separate compartments was designed for irradiation, each compartment housing one mouse. On the ninth day of the experiment, mice (without anesthesia) were exposed to whole-body X-rays at a total dose of 6 Gy. The X-ray beam was generated by a clinical linear accelerator (Siemens Primus, Germany) at a dose rate of 2 Gy/min. After exposure, the mice were removed from the Plexiglas box and returned to standard conditions. The research design is depicted in Figure 1.

### Collecting samples for laboratory testing

One day after irradiation, half of the animals (four mice per group) were sacrificed for biochemical evaluation. The remaining animals were euthanized with an intraperitoneal injection of ketamine (50 mg/kg) and xylazine (5 mg/kg) 1 week after radiation exposure for histological and immunohistochemical evaluations. The abdominal cavity was opened, and the ileum was removed, washed with phosphate-buffered saline (PBS), and homogenized in mannitol buffer (mannitol 11.64 g, sucrose 6.4 g, and EDTA 0.009 g in 1 l of water) at 4°C using a variable-speed homogenizer (10000 rpm, Heidolph, Germany). For histological and immunohistochemical analysis, the ileum was fixed in 10% neutral buffered formalin. The design of this study is summarized in Figure 1.

### Biochemical assays

Homogenized ileum tissues were centrifuged at 4°C (1000 rpm), and the supernatants were collected for measuring oxidative stress biomarkers. The total protein content of the supernatants was determined using the Coomassie blue method as described by Bradford (Spector 1978). Protein content (mg) for all samples was adjusted and diluted with Tris buffer (Tris-HCl 0.079 g, Na_2_HPO_4_ 0.268 g, MgCl_2_ 0.019 g, KCl 0.149 g, sucrose 8.9 g in 1 l of water).

### Lipid peroxidation level determination

Malondialdehyde (MDA) levels were measured using the thiobarbituric acid (TBA) method in ileum supernatants. Briefly, 200 μl of homogenized supernatant, 200 μl of 85% phosphoric acid, and 25 μl of TBA (0.04 g in 10 ml water) were mixed, then heated at 95°C for 45 min, cooled in an ice bath for 10 min. After adding n-butanol (500 μl), the mixture was centrifuged at 4°C (10 min, 10000 × g). The absorbance was read at 595 nm by a plate reader machine. MDA concentrations were expressed as μM (*n* = 4) using tetra methoxy propane as a standard (Taghizadeh et al., 2020; Taghizadeh et al., 2021).

### GSH evaluation

Glutathione (GSH) levels were measured using the 5,5’-dithiobis-(2-nitrobenzoic acid) (DTNB) reagent in ileum supernatants. The reaction between thiol and DTNB produces a yellow color, and absorbance was read at 412 nm. Results were presented in μM (Hamzeh et al. 2018).

### Protein carbonyl evaluation

Protein carbonyl (PC) levels were measured using 2,4-dinitrophenylhydrazine (DNPH) reagent in ileum supernatants. In this assay, 200 μl of sample supernatant was mixed with 300 μl of DNPH, and the mixture was heated in a water bath at 37°C for 30 min, then centrifuged at 4°C (10 min at 10000 × g). After washing with ethanol–ethyl acetate (500 μl), the mixture was centrifuged again at 4°C (10 min at 10000 × g). The precipitated proteins were dissolved in 600 μl guanidine hydrochloride, and then heated at 37°C for 15 min, and centrifuged at 4°C (10 min at 10000 × g). The absorbance of samples was read at wavelength 375 nm. Ileum protein carbonyl content was calculated as mM (Dalle-Donne et al., 2003).

### Histopathological examination of ileum tissue

Ileum tissues were fixed in 10% buffered formalin, dehydrated with alcohol, cleared with xylene, embedded in paraffin, and sectioned into 5 μm slices. Sections were stained with hematoxylin and eosin (H&E) (Naeimi et al., 2017). Inflammatory cell infiltration, hemorrhage, congestion, and vacuolation were examined in epithelial layer cells. Tissue sections were evaluated with light microscope (Nikon; Tokyo, Japan) by a histologist blinded to the research groups. For semiquantitative evaluation, prepared slides were evaluated for ileum injury using a scoring system. Given the extent and severity of ileum injury for each field, morphology, inflammatory cell infiltration, congestion, desquamation of epithelial cells, ileum damage was scored on a scale: 0 (normal ileum), 1 (slight), 2 (mild), 3 (moderate), and 4 (severe) (Deniz et al. 2015). In the semiquantitative evaluation, eight slides from each sample and five fields of each slide were evaluated with a light microscope (Nikon; Tokyo, Japan) and then, the average scores were considered for each ileum sample in the groups.

### Immunohistochemical examination of ileum tissue

For caspase-3 evaluation, deparaffinized slides were rehydrated in the graded ethanol fractionation (from absolute to 70%). Peroxide blocking was performed with 0.3% H_2_O_2_ (in methanol, room temperature, 15 min). After incubation with primary antibodies (anticaspase-3, rabbit polyclonal, 1 : 100 in TBS, v/v, 4°C overnight). Slides were then incubated with secondary antibody (Mouse and Rabbit Specific HRP/DAB) for 20 min. Then, slides were incubated with diaminobenzidine tetrahydrochloride for 5 min, then were dehydrated and mounted (Xu et al., 2014; Naeimi et al., 2017). Subsequently, all the samples were assessed with a microscope with a magnification of × 40. For quantitative analysis, immunohistochemical micrographs were assessed using ImageJ software (MacBiophotonics, version 1.41a) by densitometry. Staining intensity was determined as the percentage of the stained area to the entire surface.

### Statistical analysis

Data were analyzed using GraphPad Prism software (Graph Pad Software Inc. Version 6, USA). All data are presented as mean ± SD. Statistical analysis was performed using one-way ANOVA followed by Tukey posttest. *P* < 0.05 was considered statistically significant.

## Results

### Effects of GLZ and/or IR on oxidative stress markers in ileum

**Fig. 2:**
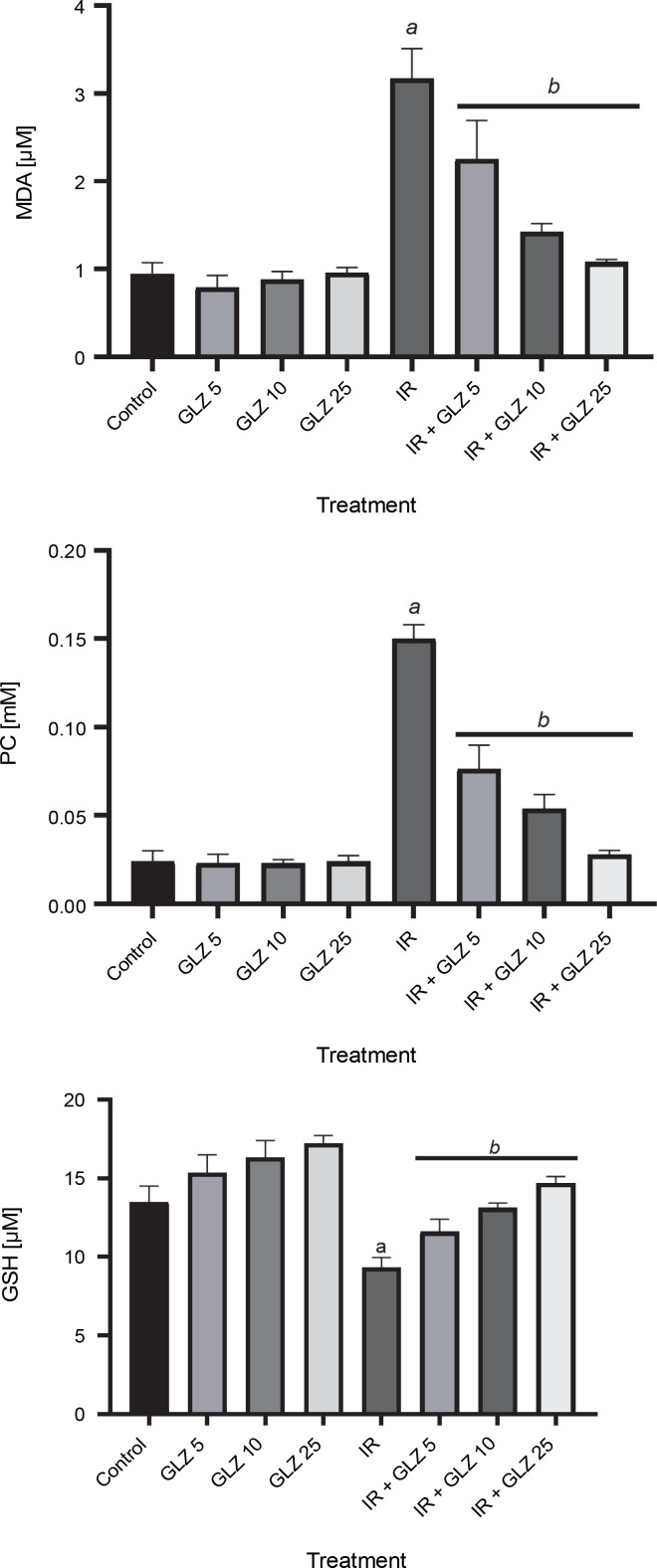
Malondialdehyde (MDA), protein carbonyl (PC), and GSH levels in the ileum of mice; mice in the IR group showed an increase in the MDA and PC and a decrease in GSH content compared with the control group; pretreatment with gliclazide (GLZ) in IR (irradiation) exposed mice significantly decreased the concentration of MDA and PC and increased GSH content in the ileum tissues compared with the IR group; all values are expressed as mean ± SD; ^a^ significant vs. control group (*P* < 0.0001), ^b^ significant versus the IR group (*P* < 0.0001)

In irradiated mice, MDA and PC levels increased by 3.3 and 6.2 times, respectively, compared to control mice (*P* < 0.0001) (Fig. 2), while GSH levels decreased by 1.4 times (*P* < 0.0001). The MDA and PC levels were significantly decreased in irradiated mice when pretreated with GLZ at three tested doses as compared to the IR alone group by 1.5, 2.1, and 2.7 times for MDA, and 1.4, 2.2, and 2.9 times for PC, respectively (*P* < 0.0001). The GSH content was significantly increased when IR mice were pretreated with GLZ at three tested doses (*P* < 0.0001) as compared with IR alone mice by 1.2, 1.4, and 1.6 times, respectively.

### Effects of GLZ on ileum damage through histopathology observations

**Fig. 3:**
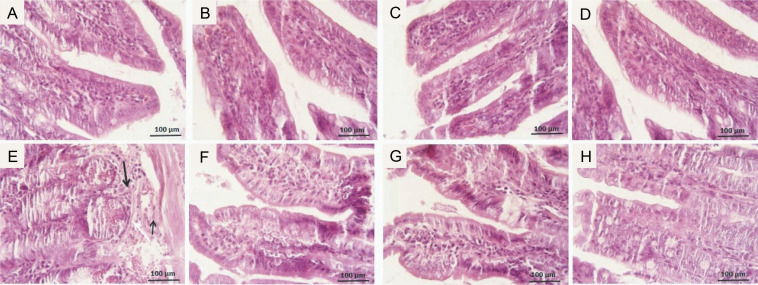
Micrographs of the ileum tissues in A) control group with regular epithelial layer and goblet cells; B, C, D) GLZ treatment groups at three doses of 5, 10, and 25 mg/kg; E) IR-exposed group with epithelial cell detachment, hemorrhage (arrow narrow), and inflammatory cell infiltration (thick arrow) in most of the areas; F, G, H) GLZ + IR groups; H&E staining, magnification 40, scale bare, 100 μm, IR – irradiation, GLZ – gliclazide

The photomicrographs of ileum tissues in all groups are presented in Figure 3. In the control group, stained with hematoxylin and eosin, the ileum tissue structure was normal across all layers: mucosa, submucosa, muscle, and serosa. Epithelial cell shedding, rupture, hemorrhage, and infiltration of inflammatory cells into the submucosal layer were commonly seen in mice exposed to radiation. GLZ administration before irradiation reduced these negative effects. Among the three doses of GLZ administered, 25 mg/kg showed greater effectiveness in mitigating histopathological alterations compared to 5 and 10 mg/kg.

**Fig. 4:**
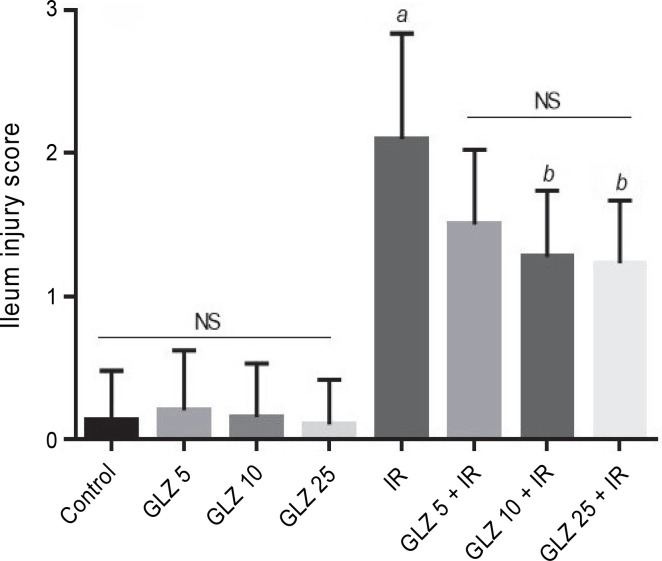
Effect of GLZ and IR on the microscopic ileum injury score; IR – irradiation, GLZ – gliclazide, NS – non-significant, between groups (control and GLZ 5, GLZ 10, GLZ 25) and (GLZ 5 + IR and GLZ 10 + IR and GLZ 25 + IR); *a* – IR and control (*P* < 0.001), *b* – GLZ 10 + IR and GLZ 25 + IR with IR (*P* < 0.05)

Microscopic scores of ileum samples were 2.1 ± 0.74 in IR-exposed mice, while in the control group, the score was 0.13 ± 0.35. However, the scores were significantly lower in the GLZ + IR groups at doses of 10 and 25 mg/kg (*P* < 0.05 and *P* < 0.01, respectively) compared to the IR-only group (Fig. 4).

### Effects of GLZ on ileum damage through immunohistochemical observations

**Fig. 5:**
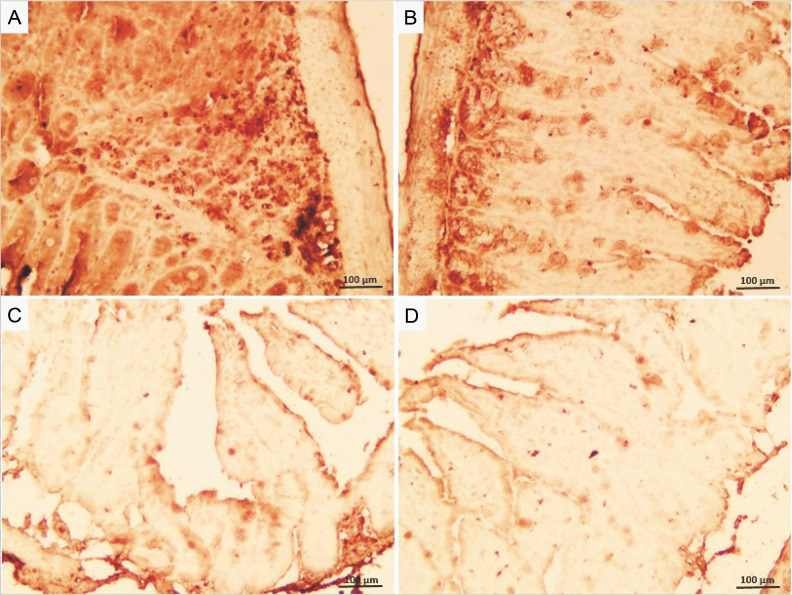
Immunohistochemical staining of caspase-3 in mice ileum: A) the irradiated group showed a significant increase in caspase-3 immunoreactivity in the ileum cells, B, C, and D) GLZ (gliclazide) (5, 10, and 25 mg/kg) + IR (Irradiation) group demonstrated a significant reduction in caspase-3 immunostaining; the brown color indicates caspase-3 positive cells (magnification ×40) because the control and GLZ groups did not show any apoptosis without any color

Irradiation increased caspase-3 expression, an indicator of apoptosis, in ileum tissue, observed as a brown color (Fig. 5). Neither the control nor GLZ groups exhibited caspase-3 expression, showing virtually no brown staining. Caspase-3 expression significantly increased in all ileum cells in IR groups compared with the control and GLZ groups at all doses. However, GLZ treatment significantly reduced caspase-3 expression in IR-exposed mice.

**Fig. 6:**
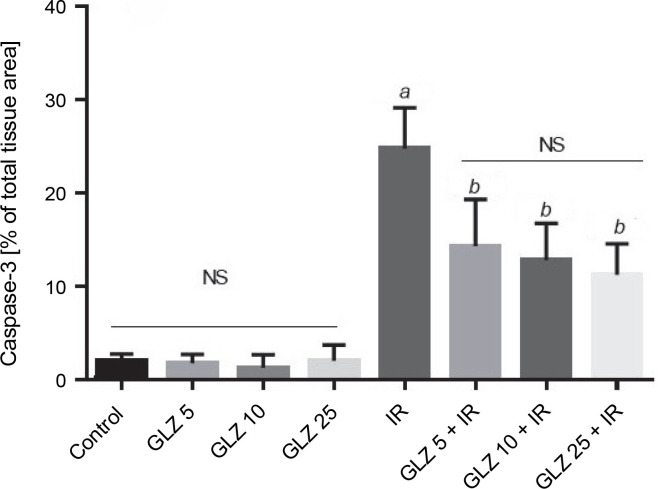
Ileum immunoreactivity scores of caspase-3 in mice; IR – irradiation, GLZ – gliclazide, NS – non-significant, between groups (control and GLZ 5, GLZ 10, GLZ 25) and (GLZ 5 + IR and GLZ 10 + IR and GLZ 25 + IR); *a* – IR and control (*P* < 0.001), *b* – GLZ 5 + IR, GLZ 10 + IR and GLZ 25 + IR with IR (*P* < 0.05)

In the semiquantification of immunostaining for caspase-3 (Fig. 6), the score of immunopositive irradiated cells was 24.76 ± 4.38, significantly higher than in the control (1.86 ± 0.88) and GLZ-treated groups. GLZ (10 and 25 mg/kg) treatment markedly decreased the apoptotic index in irradiated ileum (*P* < 0.01).

## Discussion

The intestinal tract comprises rapidly dividing cells, with the ileum’s primary function being nutrient absorption. Intestinal epithelial cells also serve as a barrier against microbial infiltration into the bloodstream. Due to the high self-renewal rate of intestinal cells, this tissue is highly sensitive to IR (Moraitis et al., 2023). This study evaluated the antioxidant and anti-apoptotic effects of GLZ in protecting against IR-induced intestinal injury in mice. GLZ administration for 8 days protected ileum tissue from IR-induced damage, reducing levels of oxidative stress biomarkers MDA and PC and caspase-3 immunoreactivity in the ileum. The 25 mg/kg dose of GLZ was more effective than 5 and 10 mg/kg in mitigating IR-induced ileum injury. It is well-documented that IR generates free radicals and ROS (Hosseinimehr et al., 2020). The increase in ROS production is the main mechanism involved in tissue injury induced by radiation exposure. Therefore, antioxidants have radioprotective effects on healthy cells and tissues (Hosseinimehr et al., 2009; Talebpour Amiri et al., 2018). IR is an important source of ROS generation that results in elevated oxidative stress in the body. The increased levels of endogenous and exogenous ROS destroy the cellular antioxidant system and stimulate the cascade of ROS production, which leads to critical macromolecule damage such as DNA and protein. Excessive cellular damage results in cell death (Dong et al., 2020).

MDA, PC, and intracellular GSH are considered to be reliable biomarkers of oxidative stress. MDA is the final product of polyunsaturated fatty acids peroxidation in the cells and it is one of the oxidative stress markers. The level of MDA associated with the degree of cellular peroxidation and damage caused by ROS (Manisaligil et al., 2022). Protein carbonylation, one of the irreversible oxidative protein modifications, is a major final byproduct of the oxidation process that occurs in the cell exposed to IR (Kim et al., 2014). GSH acts as an endogenous free radical scavenger (Wang et al., 2020). Irradiation of animals resulted in elevated MDA levels and decreased GSH content in the intestinal tissues of rats (Musa et al., 2019; Radwan and Karam, 2020). In the present study, GLZ administration resulted in a decrease in the MDA and PC levels and an increase in GSH levels in the ileum tissue of IR-treated mice, which was associated with a decrease in oxidative stress levels. Previously it has been shown that GLZ administration protected mice against liver and kidney damage induced by oxidative stress (Taghizadeh et al., 2020; Taghizadeh et al., 2021). Additionally, GLZ administration decreased diabetic nephropathy by inhibiting ROS and apoptosis in rats (Wu et al., 2012).

Recently, we showed that GLZ is more effective than ascorbic acid, a well-known antioxidant, in scavenging free radicals (diphenyl-picrylhydrazyl (DPPH) at the same concentrations. In the reducing power assay, the IC_50_ value of GLZ (19.6 μM) was lower than that of ascorbic acid (40.2 μM), which showed GLZ as a more potent compound than ascorbic acid (Pouri et al., 2019). This antioxidant activity of GLZ is probably due to the presence of azabicyclo-octyl ring (O’Brien et al., 2000; Sena et al., 2009). We previously showed that GLZ reduced the percentages of micronuclei, as a DNA damage indicator, induced by IR in human normal lymphocytes (Pouri et al., 2019). However, the radioprotective effect of GLZ against ileum injury has not been investigated yet. The present study showed that exposure to IR resulted in elevated caspase-3 activity in the ileum tissues of irradiated mice. Caspase-3 immunoreactivity was decreased in GLZ pretreated and irradiated mice as compared to alone irradiated mice. It is well documented that there is a cross-talk between elevated ROS levels and caspase-3 reactivity induced by IR that results in enhancing apoptosis of cells (Li et al., 2019). GLZ administration leads to a decrease in oxidative stress levels and caspase-3 activity diminishing side effects induced by IR in intestinal tissue. These results exhibited that the immunoreactivity score of caspase-3 activity in ileum tissues of irradiated mice with GLZ pretreatment at a dose of 25 mg/kg was significantly lower than that in the GLZ at doses 5 and 10 mg/kg.

After IR exposure, intestinal epithelia were destroyed structurally at the cellular level and microbial penetration into cells was increased. The decreasing intestinal cell proliferation and increasing inflammatory response are associated with the impairment of the intestinal barrier (Kim et al., 2020; Shim et al., 2017; Zhou et al., 2020). The elevated ROS levels by IR are associated with the inflammation process that induces side effects in irradiated tissues (Salehifar and Hosseinimehr, 2016). Exposure to IR causes the production of several inflammatory mediators such as cytokines and chemokines. These mediators can activate the pro-inflammatory cascade process in cells that results in a dysfunction of normal tissues (Najafi et al., 2018). Several studies demonstrated that anti-inflammation natural products like seabuckthorn pulp, seed oils, and baicalein were able to protect against intestinal injury induced by IR in animals (Shi et al., 2017; Jang et al., 2019; Zhou et al., 2020). Coenzyme Q10 (CoQ10) attenuated IR-induced oxidative stress by reducing lipid peroxidation increasing the catalase activity and reducing glutathione content. It also diminished the inflammation process by downregulating intestinal inflammation biomarkers (Mohamed and Said, 2021). Exposure to IR significantly decreased the levels of inflammation-related factors in mice treated with alpha-lipoic acid. The alpha-lipoic acid treatment before exposure to IR decreased the IR-induced intestinal damage by diminishing the oxidative stress level and inflammation (Jeong et al., 2016). In a previous study, GLZ markedly attenuated the proinflammatory mediator and process in healthy tissues and was able to diminish ulcerative colitis (Arafa et al., 2020). In this study, a strong cross-relation between oxidative stress biomarkers (MDA, PC, and GSH), and apoptosis markers (caspase-3) in the normal ileum in irradiated mice was observed. Whereas, exposure to IR resulted in increased MDA and PC levels as well as caspase-3 immunoreactivity in the ileum of mice. Briefly, IR generates ROS in cells and activates multiple cascades of oxidative stress and proinflammatory processes that result in intestinal damage in mice. GLZ, as an approved medicine, was able to diminish all these deleterious events in normal tissue and protect mice against ileum damage induced by IR.

## Conclusion

GLZ, with its antioxidant and anti-apoptotic properties, protected against IR-induced ileum damage by reducing oxidative stress and caspase-3 immunoreactivity in irradiated mice. GLZ treatment improved ileum tissue structure in irradiated mice, suggesting a potential radioprotective role for GLZ in patients undergoing radiation therapy.

## Data Availability

The data sets used and/or analyzed during the current study are available from the corresponding author upon reasonable request.

## Authors’ information

S.A. is a Pharm D. student, S.F. is PhD in radiopharmacy, F.T.A. is an associate professor in anatomy and histology, and S.J.H. is a professor in radiopharmacy.
