# Erratum to the article: „Gliclazide protects ionizing radiationinduced intestinal injury in mice by inhibiting oxidative stress and caspase-3”. BioTechnologia 2024; 105(4): 367-376. DOI: https://doi.org/10.5114/bta.2024.145257

**DOI:** 10.5114/bta/237639

**Published:** 2026-09-27

**Authors:** Soroush Arzani, Soghra Farzipour, Fereshteh Talebpour Amiri, Seyed Jalal Hosseinimehr

## Erratum notice

The authors have advised that there is an error in the Figure 3. The image in panel B was inadvertently duplicated, resulting in panels B and D appearing identical in the published figure.

In the previously published version of the article, the following Figure 3 was presented:

**Figure:**
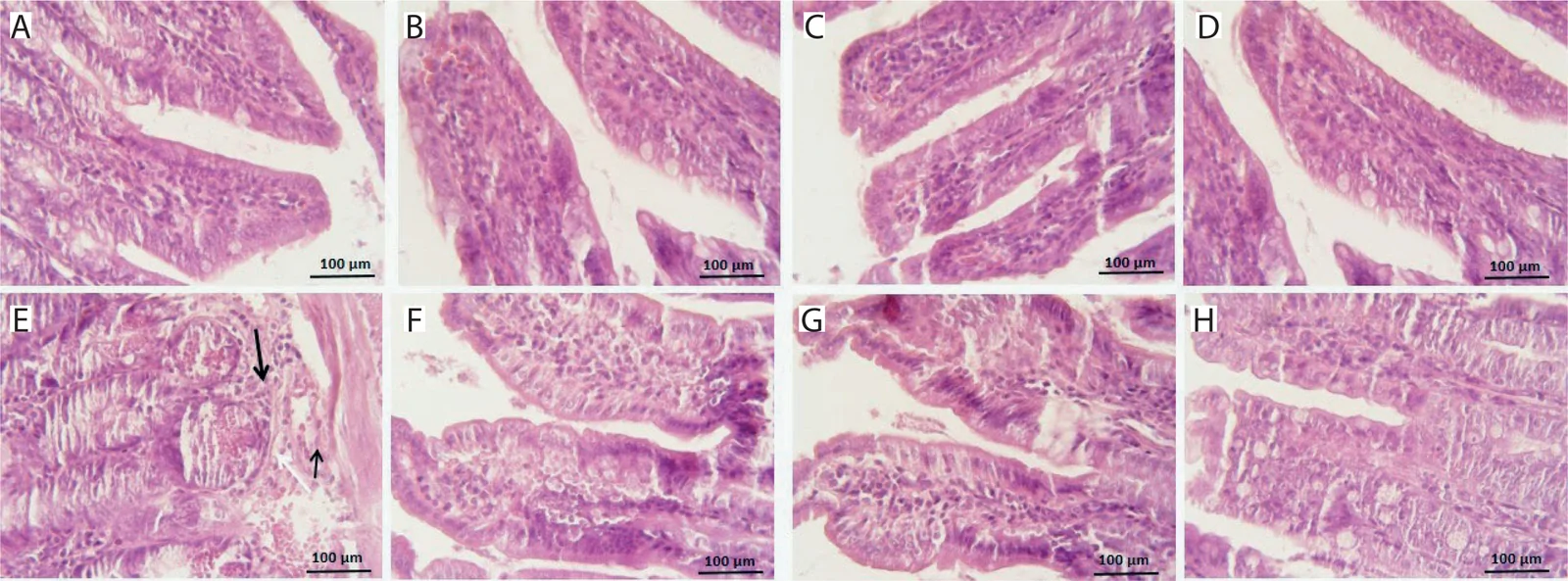


This has been corrected to:

**Figure:**
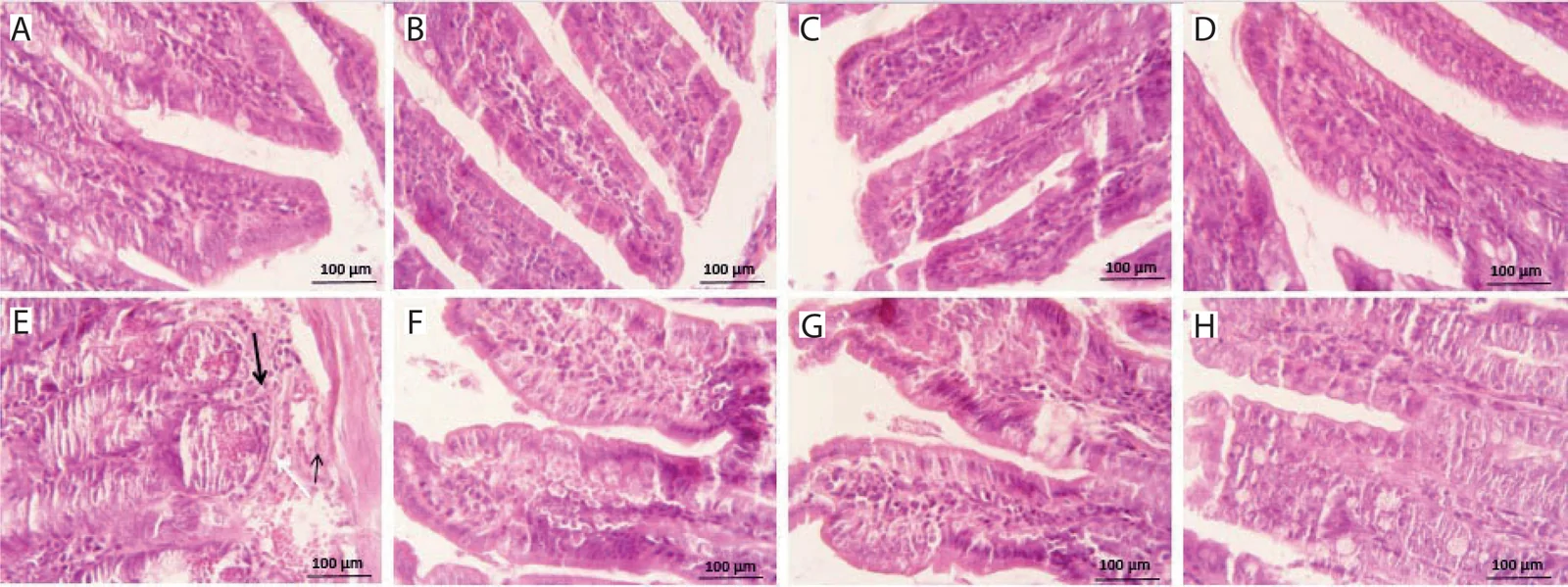


Erratum to the article is also applied directly in the digital file posted on the journal’s website.

